# Catheter-Related *Staphylococcus aureus* Bacteremia and Septic Thrombosis: The Role of Anticoagulation Therapy and Duration of Intravenous Antibiotic Therapy

**DOI:** 10.1093/ofid/ofy249

**Published:** 2018-10-01

**Authors:** Rita Wilson Dib, Anne-Marie Chaftari, Ray Y Hachem, Ying Yuan, Dima Dandachi, Issam I Raad

**Affiliations:** 1 Department of Infectious Diseases, Infection Control & Employee Health, The University of Texas MD Anderson Cancer Center, Houston, Texas; 2 Section of Infectious Diseases, Department of Medicine, Baylor College of Medicine, Houston, Texas

**Keywords:** anticoagulation, bacteremia, catheter-related bloodstream infection, septic thrombophlebitis, *Staphylococcus aureus*

## Abstract

**Background:**

Catheter-related septic thrombosis is suspected in patients with persistent central line–associated bloodstream infection (CLABSI) after 72 hours of appropriate antimicrobial therapy. The clinical diagnosis and management of this entity can be challenging as limited data are available. We retrospectively studied the clinical characteristics of patients with *Staphylococcus aureus* catheter-related septic thrombosis and the outcomes related to different management strategies.

**Methods:**

This retrospective study included patients with CLABSI due to *S. aureus* who had concomitant radiographic evidence of catheter site thrombosis treated at our institution between the years 2005 and 2016. We collected data pertaining to patients’ medical history, clinical presentation, management, and outcome within 3 months of bacteremia onset.

**Results:**

A total of 128 patients were included. We found no significant difference in overall outcome between patients who had deep vs superficial thrombosis. Patients with superficial thrombosis were found to have a higher rate of pulmonary complications (25% vs 6%; *P* = .01) compared with those with deep thrombosis. Patients who received less than 28 days of intravascular antibiotic therapy had higher all-cause mortality (31 vs 5%; *P* = .001). A multivariate logistic regression analysis identified 2 predictors of treatment failure: ICU admission during their illness (odds ratio [OR], 2.74; 95% confidence interval [CI], 1.08–6.99; *P* = .034) and not receiving anticoagulation therapy (OR, 0.24; 95% CI, 0.11–0.54; *P* < .001).

**Conclusions:**

Our findings suggest that the presence of *S. aureus* CLABSI in the setting of catheter-related thrombosis may warrant prolonged intravascular antimicrobial therapy and administration of anticoagulation therapy in critically ill cancer patients.

Catheter-related septic thrombosis is typically suspected in patients with persistent bacteremia after 72 hours of appropriate antimicrobial therapy. The diagnosis is based on high clinical suspicion with radiographic evidence of thrombosis [1]. Compared with the general population, cancer patients are at higher risk of catheter-related bacteremia complications [2–4].

There is a bidirectional association between bacteremia, intravascular catheters, and the occurrence of venous thrombosis. Long-term indwelling catheters are known to independently increase the risk of venous thrombosis because the insertion can cause damage to the vessel wall and activation of the intrinsic coagulation pathway [5]. Seventy percent of secondary upper extremity deep venous thrombosis events occur in the presence of central venous catheters [6]. On the other hand, septicemia has been shown to be associated with a ≥3-fold increase in the risk of development of deep venous thrombosis [7]. This is particularly true in cancer patients; thrombotic complications have been shown to be common in catheterized veins of cancer patients and are often associated with central line–associated bloodstream infection (CLABSI) [8]. Our previous studies show that in cancer patients with CLABSI caused by *Staphylococcus aureus,* septic thrombophlebitis is 1 of the most common manifestations of a deep-seated infection, occurring in 15%–24% of cases [3, 9].

Septic thrombosis is defined as infection of a thrombus. However, it is challenging to differentiate between the simultaneous presence of catheter-related thrombosis and bacteremia and the actual suppuration of the thrombus that is causing the septic thrombosis. Additionally, there are limited data guiding the adequate duration and route of antimicrobials, particularly the proper duration of intravenous (IV) antibiotics. The current guidelines are not based on high-quality evidence. Furthermore, the role of anticoagulants in the management of septic thrombosis is unclear.

We sought to evaluate the clinical characteristics of *S. aureus* catheter-related septic thrombosis, appropriate management, optimal duration of IV treatment, and the role of anticoagulation therapy.

## METHODS

### Study Design and Patient Population

In this retrospective study, we reviewed the medical records of all patients with *S. aureus* CLABSI treated at The University of Texas MD Anderson Cancer Center from December 2005 to December 2016.

CLABSI is defined as a laboratory-confirmed bloodstream infection (not related to an infection at another site) where a central line has been in place for at least 48 hours before the development of bacteremia [10]. Only patients who had concomitant radiographic evidence of thrombosis along the catheter around the time of *S. aureus* bloodstream infection were included. We excluded patients with lower extremity venous thrombosis in order to preserve the homogeneity of the data set. The study was approved by the MD Anderson Institutional Review Board. Waiver of informed consent for this study was obtained.

### Study Variables

We extracted from the medical records demographic variables, preexisting medical conditions such as underlying malignancy at the time of diagnosis and history of hematopoietic stem cell transplantation (HSCT), and medication administration records within 30 days, including chemotherapy, radiation therapy, or steroids. We collected data on the clinical presentation, including fever, local signs of inflammation, sepsis, and whether the patient required admission to the intensive care unit (ICU) at any point during their illness. Neutropenia is defined as an absolute neutrophil count (ANC) of <500 cells/µL at the time of bacteremia. Clinical presentation of sepsis was defined according to the previous 2001 SCCM/ESICM/ACCP/ATS/SIS International Sepsis Definitions Conference guidelines [11].

We also reviewed the catheter type, location, and number of lumens. We further divided the patients into 2 subgroups, those with superficial vs deep venous thrombosis, based on the anatomical classification of the affected veins in the upper extremities. A thrombus that extended between a superficial and a deep vein was classified with the deep venous thrombosis group. We conducted a subset analysis comparing the baseline characteristics and outcomes of the superficial and deep venous thrombosis groups.

We reviewed the choice of antimicrobial therapy, the duration of IV and oral therapy, and whether the patients received anticoagulation therapy. The total duration of effective antimicrobial therapy, based on in vitro susceptibility, was calculated in days. For further analysis, the duration of IV antibiotic therapy was dichotomized as a short (14–27 days) or long course (>28 days).

### Study Outcomes

We evaluated patients for fever resolution and microbiologic eradication within 72 hours. For afebrile patients upon presentation, we relied solely on microbiologic data. The primary outcome was success or failure at the end of therapy. Failure was defined as the presence of at least 1 of the following, and success was defined as the absence of all of the following:

• persistence of fever, which was considered present if a patient’s recorded temperature was ≥100.4^○^F (38^○^C) beyond 7 days while the patient was receiving appropriate antimicrobial therapy;
• persistence of bacteremia, defined as positive blood culture for the same organism in a sample drawn beyond 7 days of appropriate antimicrobial therapy;
• relapse or recurrence of bacteremia within 3 months;
• new complication (including septic arthritis, deep tissue abscess, epidural abscess, psoas abscess, meningitis, osteomyelitis, or septic pulmonary emboli) related to the bacteremia that was not present at onset and was discovered after the end of IV antibiotic therapy;
• all-cause mortality within 3 months of bacteremia.

### Statistical Analysis

Categorical variables were compared using the chi-square test or Fisher exact test, as appropriate. Continuous variables were compared using the Wilcoxon rank-sum test. Logistic regression analysis was used to identify the factors that were independently associated with the overall outcome of success vs failure. All the tests were 2-sided tests with a significance level of .05. The data analyses were performed using SAS, version 9.3 (SAS Institute Inc., Cary, NC).

## RESULTS

### Patient Characteristics and Baseline Information

A total of 128 patients met the inclusion criteria, of whom 81 were male (63%). The median age (range) was 55 (4–88) years. The underlying malignancy was hematological in 66 patients (52%). Neutropenia was present in 29 (23%) of the patients at the time of bacteremia. The thrombosis was diagnosed using Duplex ultrasonography in 96%, contrast venography in 3%, and computed tomography with contrast in 1% of the patients.

For 74% of patients, multiple positive blood cultures taken on the same or different dates had been recorded. At presentation, 101 patients (79%) had fever and 79 (62%) had sepsis. In 55% (71/128) of patients, local signs indicative of thrombosis were present, manifesting primarily as erythema (27%), induration (20%), and tenderness (16%).

Forty-seven percent of infections were due to methicillin-resistant *S. aureus* (MRSA). Seven patients had polymicrobial infections (5%).

The majority of the catheters were either removed (94%) or exchanged (4%). We found that 63% of the patients had their catheters removed or exchanged within 2 days. The most frequently used antimicrobial was vancomycin (84%), followed by daptomycin (47%), cefepime (32%), and linezolid (25%) monotherapy. Unless contraindicated because of underlying allergy, methicillin-sensitive *Staphylococcus aureus* (MSSA) infections were treated with nafcillin in 38% of the cases after identification of susceptibility. The median duration of IV antimicrobial treatment (range) was 24 (1–69) days, and the median total duration of IV and oral therapy was 30 (3–161) days. Anticoagulation therapy was administered in 88 patients (69%); the majority received enoxaparin (83%). The median duration of anticoagulation (range) was 91 (1–500) days. More than 90% of these patients were treated with anticoagulation for more than 1 week.

Microbiological eradication was achieved in 98% of the patients. Ninety-four percent (94/100) achieved defervesnece. Relapse or recurrence occurred in 8% of the patients. Sixteen percent of the patients died, with 2 deaths deemed related to bacteremia by their attending physicians.

### Fever and Microbiologic Resolution

Patients who achieved defervescence and microbiologic eradication within 72 hours of bacteremia onset were compared with patients with persistent fever or bacteremia beyond 72 hours. Both groups had similar demographics and clinical characteristics (Table 1). The only intervention that had an association trend with fever/microbiologic resolution at 72 hours was central venous catheter removal or exchange within 48 hours of the onset of bacteremia. Seventy-four percent of patients had their catheter removed or exchanged within 48 hours vs 57% (*P* = .09) (Table 1). The median durations of IV and total antibiotic therapy were longer in the setting of persistent fever or bacteremia, although not significant (31 vs 20 days for IV; *P* = .08; 33 vs 25 days total; *P* = .17). Deep-seated infection, relapse, and mortality rates were not statistically different based on persistence of fever or bacteremia.

**Table 1.** Comparison of Patients With and Without Fever and Microbiologic Resolution Within 72 Hours

Characteristics	Both Fever and Microbiology Resolution Within 72 h		
	Yes (n = 34)	No (n = 81)	*P* Value
Age, median (range), y	51 (4–79)	56 (7–88)	.14
Male sex, No. (%)	21 (62)	53 (65)	.71
Underlying condition, No. (%)			.17
Hematological malignancy	15 (44)	47 (58)	
Solid tumor	19 (56)	34 (42)	
Risk factors (within 30 d of bacteremia), No. (%)			
Chemotherapy	27 (79)	61 (75)	.64
Steroids	10 (29)	28 (35)	.59
Radiation	4 (12)	7/79 (9)	.73
Surgery	2 (6)	3 (4)	.63
MRSA, No. (%)	16 (47)	40 (49)	.82
ANC <500 cells/µL at bacteremia, No. (%)	8 (24)	19 (23)	.99
Catheter type, No. (%)			.59
PICC	20 (59)	55 (68)	
Central venous catheter	11 (32)	17 (21)	
Surgically implanted (port-a-cath)	3 (9)	9 (11)	
Catheter lumens, No. (%)			.19
Single	5 (15)	19/79 (24)	
Double	26 (76)	58/79 (73)	
Triple	3 (9)	2/79 (3)	
Clinical presentation, No. (%)			
Fever (temperature ≥38°C)	24 (71)	67 (83)	.14
Sepsis on presentation	15 (44)	55 (68)	.06
Required ICU admission	6 (18)	17 (21)	.68
Local signs of inflammation	19 (56)	44 (54)	.88
Management			
Catheter removed/exchanged, No. (%)	33 (97)	81 (100)	.3
Catheter removal/exchange within 2 d of bacteremia, No. (%)	25 (74)	46 (57)	.09
Duration of IV antibiotic therapy, median (range), d	20 (3–49)	31 (1–69)	.08
Total duration of antibiotic (IV and oral), median (range), d	25 (6–62)	33 (3–161)	.17
Anticoagulation, No. (%)	25 (74)	54 (67)	.47
Outcome, No. (%)			
Development of deep-seated infection	6 (18)	17^a^ (21)	.68
Relapse/recurrence within 3 mo	0 (0)	6/75 (8)	.17
Mortality within 3 mo	4 (12)	11/78 (14)	>.99

Abbreviations: ANC, absolute neutrophil count; ICU, intensive care unit; IV, intravenous; MRSA, methicillin-resistant *Staphylococcus aureus*; PICC, peripherally inserted central catheter.

^a^One patient had 3 deep-seated infections: septic arthritis, deep tissue abscess, and osteomyelitis.

### Deep vs Superficial Thrombosis

There were 20 patients with superficial and 108 with deep venous thrombosis (Table 2). By univariate analysis, the baseline characteristics and clinical presentation were similar in both groups. The catheter type and number of lumens did significantly influence the location of the thrombus formation.

**Table 2.** Comparison of Patients with Superficial vs Deep Venous Thrombosis

Characteristic	Superficial Thrombosis (n = 20)	Deep Thrombosis (n = 108)	*P* Value
Age, median (range), y	54 (4–67)	55 (7–88)	.55
Male sex, No. (%)	11 (55)	70 (65)	.40
Underlying condition, No. (%)			.41
Hematological malignancy	12 (60)	54 (50)	
Solid tumor	8 (40)	54 (50)	
Risk factors (within 30 d of bacteremia), No. (%)			
Chemotherapy	18 (90)	81 (75)	.24
Steroids	9 (45)	33 (31)	.21
Radiation	0 (0)	14/106 (13)	.12
Surgery	0 (0)	6 (6)	.59
MRSA, No. (%)	11 (55)	49 (45)	.43
ANC <500 cells/µL at bacteremia, No. (%)	8 (40)	21 (19)	.08
Catheter type, No. (%)			.96
PICC	13 (65)	73 (68)	
Central venous catheter	5 (25)	25 (23)	
Surgically implanted (port-a-cath)	2 (10)	10 (9)	
Catheter lumens, No. (%)			.32
Single	2/19 (11)	26/107 (24)	
Double	17/19 (89)	76/107 (71)	
Triple	0/19 (0)	5/107 (5)	
Clinical presentation, No. (%)			
Fever (temperature ≥38°C)	19 (95)	82 (76)	.07
Type of sepsis on presentation	11 (55)	68 (63)	.96
Required ICU admission	5 (25)	22 (20)	.77
Local signs of inflammation	10 (50)	61 (56)	.59
Management			
Duration of IV antibiotic therapy, median (range), d	27 (1–49)	24 (1–69)	.83
Total duration of antibiotic (IV and oral), median (range), d	33 (14–62)	29 (3–161)	.27
Catheter removed/exchanged, No. (%)	20 (100)	105 (97)	>.99
Time to first negative blood culture, median (range), d	3 (1–14)	4 (1–16)	.11
Time to fever resolution, median (range), d	5 (1–14)	2 (1–11)	<.001
Anticoagulation, No. (%)	9 (45)	79 (73)	.013
Outcome, No. (%)			
Development of deep-seated infection	7 (35)	17^a^ (16)	.06
Septic arthritis	0 (0)	1 (1)	>.99
Deep tissue abscess	1 (5)	4 (4)	.58
Osteomyelitis	0 (0)	1 (1)	>.99
Pulmonary complications	5 (25)	6 (6)	.01
Chest wall abscess	0	1 (1)	>.99
Endocarditis	1 (5)	6/105 (6)	>.99
Overall outcome,^b^ No. (%)			.63
Success	11 (55)	65/107 (61)	
Failure	9 (45)	42/107 (39)	

Abbreviations: ANC, absolute neutrophil count; ICU, intensive care unit; IV, intravenous; MRSA, methicillin-resistant *Staphylococcus aureus*; PICC, peripherally inserted central catheter.

^a^One patient had 3 deep seated infections: septic arthritis, deep tissue abscess, and osteomyelitis.

^b^One patient was nonevaluable for overall outcome due to being lost to follow-up.

We noted that the median time to fever resolution was longer in the superficial venous thrombosis group (5 days vs 2 days for deep venous thrombosis; *P* < .001). In addition, the superficial group had a higher rate of pulmonary complications (mostly manifesting as septic pulmonary emboli; 25% vs 6%; *P* = .01). The deep venous thrombosis group, however, was more likely to have received anticoagulants (73% vs 45%; *P* = .013). None of the patients in the superficial thrombosis group with deep seated infections had received anticoagulation. The median total durations of antibiotic treatment were similar in both groups (29 days in superficial vs 33 days in deep venous thrombosis; *P* = .27) There was no significant difference between the 2 groups in terms of relapse, mortality, and overall outcome.

### Duration of Intravenous Antibiotic Therapy

We next compared patients with short duration (14–27 days) vs prolonged duration (≥28 days) of IV antimicrobial treatment. For this analysis, we excluded 20 patients who had either early-onset complications or died within 14 days of bacteremia diagnosis. This subanalysis included 36 patients who received the shorter IV antimicrobial treatment course and 45 who received the prolonged IV course. The rate and duration of subsequent oral therapy were similar in both groups. The median duration of bacteremia was significantly shorter in patients who received a shorter duration of IV antibiotic therapy (3 vs 5 days; *P* = .001).

Fever resolution, development of deep-seated infections, and relapse within the 3-month follow-up period showed no significant difference between the compared groups. The overall mortality rate, however, was significantly lower in the group of patients who received ≥28 days of appropriate IV antimicrobial therapy (5% vs 31%; *P* = .001) (Table 3), which was confirmed by a multivariate logistic regression analysis (data not shown).

**Table 3.** Comparison of Patients With Different Durations of IV Antibiotic Treatment: Subset Analysis^a^

Variable	IV Antibiotic Treatment		*P* Value
	14–27 d (n = 36), No. (%)	≥28 d (n = 45), No. (%)	
Age, median (range), y	53 (4–79)	55 (23–86)	.84
Male sex, No. (%)	20 (56)	31 (69)	.22
Underlying condition, No. (%)			.18
Hematological malignancy	17 (47)	28 (62)	
Solid tumor	19 (53)	17 (38)	
MRSA, No. (%)	20 (56)	24 (53)	.84
Neutropenia (ANC <0.5) at bacteremia, No. (%)	11 (31)	10 (22)	.40
Fever (temperature ≥38°C), No. (%)	24 (67)	37 (82)	.11
Sepsis on presentation, No. (%)	20 (56)	29 (64)	.42
Required ICU stay, No. (%)	8 (22)	6 (13)	.29
Local signs of inflammation, No. (%)	18 (50)	31 (69)	.11
Type of thrombosis,^b^ No. (%)			.20
Deep veins	29 (81)	41 (91)	
Superficial veins	7 (19)	4 (9)	
Catheter removal/exchange within 2 d of bacteremia, No. (%)	20 (56)	24 (53)	.84
Oral antibiotic treatment, No. (%)	14 (39)	14 (31)	.46
Total duration of oral antibiotic treatment, median (range), d	15 (3–42)	15 (4–111)	.63
Anticoagulation, No. (%)	13 (36)	10 (22)	.17
Time to fever resolution, median (range), d	3 (1–6)	3 (1–11)	.75
Duration of bacteremia, median (range), d	3 (1–12)	5 (1–16)	.001
Outcome (within 3 mo follow-up), No. (%)			
Deep-seated infection after treatment	0 (0)	1 (2)	>.99
Relapse	3/34 (9)	3/45 (7)	>.99
Mortality	11/35 (31)	2/44 (5)	.001

Abbreviations: ANC, absolute neutrophil count; ICU, intensive care unit; IV, intravenous; MRSA, methicillin-resistant *Staphylococcus aureus*.

^a^Patients with deep-seated infections at baseline or during IV antibiotic therapy and patients who died within 14 days after bacteremia were excluded from the analysis.

^b^Patients who had both deep and superficial venous thrombosis were included in the deep venous thrombosis group.

### Predictors of Outcome

To depict the predictors of the overall outcome of these patients, we compared patients whose outcome was success with those whose outcome was failure. One patient could not be assessed because of discharge to palliative care and no recorded follow-up (Table 4).

**Table 4.** Analysis of the Predictors Associated With Therapy Success vs Failure

Characteristic	Univariate Analysis			Multivariate Analysis	
	Success (n = 76)	Failure (n = 51)	*P* Value	OR (95% CI)	*P* Value
Age, median (range), y	54 (4–80)	56 (7–88)	.19		
Male sex	53 (70)	28 (55)	.09		
Race/ethnicity			.002		
White	47 (62)	17 (33)			
African American	10 (13)	5 (10)			
Hispanic	8 (11)	8 (16)			
Other	11 (14)	21 (41)			
Type of cancer			.019		
Hematological malignancy	33 (43)	33 (65)			
Solid tumor	43 (57)	18 (35)			
Steroids within 30 d of bacteremia	18 (24)	24 (47)	.006		
*S. aureus* methicillin susceptibility			.004		
MRSA	28 (37)	32 (63)			
MSSA	48 (63)	19 (37)			
Neutropenia (ANC <500 cells/µL) at bacteremia	9 (12)	20 (39)	<.001		
Platelet count <50 at bacteremia	14 (18)	24 (47)	<.001		
Required ICU admission	10 (13)	17 (33)	.006	2.74 (1.08–6.99)	.034
Catheter removal/exchange within 2 d of bacteremia	53 (70)	26 (51)	.033		
Deep-seated infections before or during IV antibiotic therapy	10 (13)	11 (22)	.21		
Time between bacteremia and imaging test, median (range), d	3 (–4 to 45)	4 (–4 to 34)	.07		
Type of thrombosis			.63		
Deep veins	65 (86)	42 (82)			
Superficial veins	11 (14)	9 (18)			
Anticoagulation	62 (82)	25 (49)	<.001	0.24 (0.11–0.54)	<.001

One patient was excluded from analysis as he was nonevaluable for the outcome due to being lost to follow-up. The probability modeled in multivariate analysis was for “Failure” of the outcome. Only factors that were independently associated with the outcome (*P* < .05) by univariate analysis were shown in multivariate analysis.

Abbreviations: ANC, absolute neutrophil count; CI, confidence interval; ICU, intensive care unit; IV, intravenous; MRSA, methicillin-resistant *Staphylococcus aureus*; MSSA, methicillin-sensitive *Staphylococcus aureus*; OR, odds ratio.

Success was achieved in 76 patients. By univariate analysis, patients with failures were more likely to have hematologic malignancies than solid tumors (65% vs 43%; *P* = .019) and MRSA than MSSA bacteremia (63% vs 37%; *P* = .004). Patients in whom therapy failed were also more likely to have been admitted to the ICU (33% vs 13%; *P* = .006). The type of thrombosis, catheter location, management, and antibiotic therapy duration did not significantly affect the overall outcome at the end of therapy. Patients with success were more likely to have received anticoagulation therapy than those with failure (82% vs 49%; *P* < .001).

By multivariate logistic regression analysis, failure was more likely if patients required admission to the ICU at any point during their illness (odds ratio [OR], 2.74; 95% confidence interval [CI], 1.08–6.99; *P* = .034) and less likely if patients received anticoagulation therapy (OR, 0.25; 95% CI, 0.11–0.57; *P* < .001).

## DISCUSSION

In this study, the results we found might pose a challenge to prior beliefs and practices pertaining to the clinical characterization and management of septic thrombosis. In particular, our results suggest that at least 28 days of active IV antimicrobial therapy and anticoagulation therapy are key to improve patients’ outcome.

The primary concern in the setting of venous thrombosis is the potential occurrence of septic emboli and metastatic infections. In our study population, the higher rate of pulmonary complications observed in the superficial venous thrombosis group was unexpected as superficial venous thrombosis is not thought to be highly associated with embolization [12]. In current practice, anticoagulation therapy for superficial thrombosis is not mandated and is only given in cases in which the thrombus is at high risk of extension or progression to deep venous thrombosis [13]. However, in this study, all the patients who had a superficial venous thrombus and developed deep-seated infections did not receive any concomitant anticoagulants. The significance of anticoagulation even in the superficial venous thrombosis subset is further highlighted in the multivariate analysis, where anticoagulation therapy remained statistically significant, favoring successful treatment even after controlling for other confounding factors such as severe neutropenia and thrombocytopenia.

Current guidelines are ambivalent when it comes to anticoagulation in the management of suppurative catheter-related thrombosis, particularly in the case of an associated superficial venous thrombosis [1]. A systematic review by Falagas and colleagues showed a lower mortality and faster defervescence in patients with septic thrombosis who received anticoagulation therapy [14]. Anticoagulation was also associated with successful treatment in some independent case series of patients with deep venous thrombosis [15, 16]. Our data confirm these findings.

Persistence of signs and symptoms of bacteremia beyond 72 hours despite appropriate antimicrobial therapy has been the driver for an extensive workup in patients with bacteremia, particularly *S. aureus* therapy [1, 17]. However, our findings indicate that approximately 30% of patients who had thrombosis and CLABSI did not have persistent bacteremia or fever, and their overall outcome was comparable to that of patients with persistent signs and symptoms. The reason for the lack of persistence could be more related to the fact that the catheter was removed/exchanged within 2 days of bacteremia onset (74% of patients) in the former group, who had early resolution. A prospective study by Picardi et al. showed that routine ultrasonographic examination within 24 hours of catheter-related bacteremia onset in neutropenic patients not only uncovered the diagnosis of septic thrombophlebitis within a median of 1 day (compared with 8 days in patients for whom the diagnosis was clinically driven) but also led to improved overall survival [18]. Furthermore, patients who do not have persistence of fever or bacteremia can still have septic thrombosis with similar rates of complications and outcomes to patients who had catheter-related septic thrombosis with persistent symptoms and should be evaluated and treated similarly.

Over the last quarter century, it has become a well-established fact that a complicated course of *S. aureus* CLABSI (including septic thrombophlebitis) should be treated with a prolonged course (exceeding 2 weeks) of active antimicrobials [3, 9, 19, 20]. The 2009 Infectious Diseases Society of America guidelines recommend up to 4 to 6 weeks of antimicrobial therapy [1]. However, none of the prior studies or the recent IDSA guidelines specify the optimal duration of IV antibiotic therapy before stepping down to oral therapy. Some experts have recommended the use of at least 4 weeks of IV antimicrobial therapy [1]. Other case reports described the need for longer duration of parenteral therapy, reaching up to 6 weeks [4, 16]. However, strong supporting evidence has been lacking.

Our data showed that treating for 28 days (4 weeks) or longer with IV antibiotics did have a survival advantage over the shorter duration (14–27 days) of IV therapy followed by oral antibiotics. However, the rates of relapse and recurrence, as well as post-treatment complications, were not significantly higher in those who received 14 to 27 days of intravenous therapy. Mertz et al. also showed that IV antibiotic treatment for 2–3 weeks was adequate to ensure eradication and prevent recurrence or relapse in injection drug users. However, only 19 (53%) of the 36 patients with bacteremia had *S. aureus,* and thrombosis was not documented. Unlike our study, Mertz et al. did not evaluate the overall survival based on duration of IV antibiotic therapy [21].

To our knowledge, this is the first study to look at the management (including the optimal duration of IV antibiotic therapy and anticoagulation) of *S. aureus* catheter-related septic thrombosis in cancer patients. Our study has several limitations, however, including the retrospective nature of the analysis and the unequal numbers in the compared groups. As our study was conducted in a single center on cancer patients, the results might not be generalizable to other populations. In addition, there was a variation in the choice of antimicrobial therapy across the years and among providers and a change in diagnostic imaging practices over the decade that may have affected the outcome. Large prospective randomized studies are needed to confirm our results.

In conclusion, our findings suggest that the presence of *S. aureus* CLABSI in the setting of catheter-related thrombosis may warrant prolonged IV antimicrobial therapy (at least 28 days) regardless of the patient’s clinical progression within the first 72 hours. Moreover, serious consideration should be given to including anticoagulation in the management of *S. aureus* catheter-related venous thrombosis. A future prospective clinical trial may be needed to further validate our findings. Septic embolization may occur frequently in patients with *S. aureus* CLABSI and superficial venous thrombosis, which should be treated like CLABSI with deep venous septic thrombosis.
